# 

**Published:** 1895-11

**Authors:** 


### PERSONAL.

Veterinarian A. E. Conrow, of Burlington, N. J., has just been selected as the Democratic candidate for Assembly for that district.

Dr. Tait Butler will in his new field of journalistic work, as editor of the *Southern Farm Gazette*, contribute much toward enlarging the field of employment for veterinarians in the South. Better grades of stock mean greater risks and perils, and valuable stock cannot be left to the care of ignorant pretenders and voodoo doctors. We wish him success in his new field of labor.

Prof. A. Smith, of Toronto, has recently been confined to the house by a painful accident.

Dr. J. Payne Lowe has become identified as veterinary inspector with the Passaic City Board of Health.

Among Philadelphia's Councilmen we note the presence there of veterinarian John Z. Tintsman.

Dr. Joseph M. Good, of Chattanooga, Tennessee, has just emerged from the hospital after a serious illness.

The report of Dr. Paul Fischer's return to the Ohio State University seems to have been somewhat premature.

Dr. H. M. Ball, of Columbus, Ohio, has become associated with the Veterinary Department of the Ohio State University.

Dr. Charles McEachran officiated as one of the stewards at the Montreal Hunt Club in the early part of October.

The many friends of Dr. H. D. Gill, of the JOURNAL staff, will regret to learn of the painful injuries he recently received by being thrown from a cart, while on a suburban professional mission.

Dr. A. S. Wheeler, of New Orleans, Louisiana, is at present sojourning for a time in Europe.

Dr. W. L. Williams, of Bozeman, Montana, has suffered with a severe attack of "grippe" since his return from the Des Moines Convention.

Dr. J. R. McLaughlin, veterinarian, occupies the position of Inspector of Provisions, etc., for the district of Newton, Massachusetts.

Dr. W. W. James, a veterinary dentist, was recently seriously injured by a stallion, having entered the latter's stall alone for the purpose of dressing the teeth.

Dr. Charles A. Dohan, as one of the stewards, was keenly interested in the recent meet of the Rose Tree Hunt near Media, Pennsylvania.

---

MARRIED.—At Philadelphia on October 10, 1895, John J. Maher, V.M.D., of Philadelphia, to Caroline S. R. Engelhardt, of Germany.

